# The mediating role of insulin resistance in the association between inflammatory score and MAFLD: NHANES 2017–2018

**DOI:** 10.1002/iid3.70035

**Published:** 2024-10-04

**Authors:** Yan Chen, Xin Zhao

**Affiliations:** ^1^ Department of Cardiology The Second Hospital of Dalian Medical University Dalian People's Republic of China

**Keywords:** inflammation, MAFLD, mediation effect, NHANES, TyG

## Abstract

**Background:**

The association between inflammatory score, insulin resistance (IR), and metabolic‐associated fatty liver disease (MAFLD) is inconclusive.

**Objective:**

The objective of this study was to examine the relationship between the inflammatory score and MAFLD and investigate the potential mediating effect of IR (evaluated by triglyceride‐glucose index) in this association.

**Methods:**

Calculating inflammatory score was performed based on white blood cells and high‐sensitivity C‐reactive protein. The association between the inflammatory score and MAFLD was evaluated based on the weighted multifactor logistic regression model. Restricted cubic splines (RCS) were used to visualize the dose–response relationship between the inflammatory score and MAFLD. We also conducted a mediation analysis to assess the extent to which IR mediates this association.

**Results:**

Among the 1090 participants, 563 were ultimately diagnosed with MAFLD. Multivariate logistic regression results indicated a close positive association between inflammatory score and MAFLD (odds ratio = 1.235, 95% confidence interval 1.069–1.427, *p* = .007). The RCS results indicated a linear dose–response relationship between the inflammatory score and the risk of MAFLD after adjusting for potential confounding factors. Furthermore, the mediation analysis results showed that IR partially mediated the association between the inflammatory score and MAFLD (percent mediation = 33%).

**Conclusion:**

Our research results indicate that the inflammatory score is positively associated with the risk of MAFLD, and IR plays a partial mediating effect in this association.

## Graphical abstract

Inflammatory score positively associated with risk of metabolic‐associated fatty liver disease (MAFLD). Inflammatory score showed a linear dose–response relationship with the risk of MAFLD.

## 1 INTRODUCTION

The diagnostic criteria for metabolic‐associated fatty liver disease (MAFLD) were systematically defined in 2020.^1^ MAFLD emphasizes metabolic abnormalities as a key characteristic in such patients and is widespread worldwide with a combined global prevalence of 39.22% in previous studies.^2^ Patients with MAFLD had a significantly higher risk of all‐cause mortality compared to patients with nonalcoholic fatty liver disease (NAFLD),^3^, ^4^ and when combined with diabetes mellitus and cardiometabolic disorders, there was a significantly increased risk of adverse cardiovascular and cerebrovascular events, such as myocardial infarction and stroke.^5^ Although MAFLD impacts global public health less than malignancies and cardiovascular disease, it is necessary to pay attention to the potential impact of MAFLD on human health as the prevalence of metabolic syndrome and diabetes is increasing worldwide.^6^, ^7^

Levels of systemic inflammation are strongly associated with the risk of MAFLD.^8^ The inflammatory score is a novel index that reflects the overall inflammatory burden of the body, calculated by combining Z‐scores of different inflammatory biomarkers such as white blood cells (WBC), high‐sensitivity C‐reactive protein (hs‐CRP), complements C3, C4, fibrinogen, and so on.^9^ Although the methods of calculation of inflammatory score varied slightly across studies, they generally included WBC and hs‐CRP, and these studies showed that inflammatory score were strongly associated with cardiometabolic health, atherosclerotic progression, and cancer prognosis.^9^, ^10^, ^11^, ^12^, ^13^ IR also plays a significant role in the onset and progression of MAFLD,^14^ and inflammatory response and IR are often inseparable during the occurrence and development of MAFLD, which often act together in the pathogenesis of MAFLD.^15^, ^16^, ^17^, ^18^ Therefore, it is necessary to clarify the internal relationship between inflammation, IR, and MAFLD.

The triglyceride‐glucose (TyG) index, calculated based on fasting triglycerides and glucose, is another effective method for assessing IR.^19^ Compared to traditional methods such as the hyperinsulinemic‐euglycemic clamp (HIEC) and the homeostasis model assessment‐insulin resistance (HOMA‐IR), the TyG offers the advantages of low cost and easy accessibility when assessing individual IR. While HIEC is regarded as the gold standard for evaluating IR, its invasiveness and high cost make it impractical for widespread clinical use. Similarly, although HOMA‐IR offers another means of assessing IR, it necessitates fasting insulin measurements from participants and is not applicable to those using exogenous insulin or with impaired β‐cell function, thereby limiting its clinical utility. Furthermore, research indicates that the TyG index outperforms HOMA‐IR in identifying patients with metabolic syndrome.^20^ For these reasons, we consider using the TyG index to assess IR in this study.

Briefly, in this study, we used the inflammatory score to assess the overall burden of inflammation in individuals, employing TyG as a reliable surrogate for IR to clarify the intrinsic association between inflammation, IR, and MAFLD.

## 2 METHODS

### 2.1 Data source and screening

**Figure 1:**
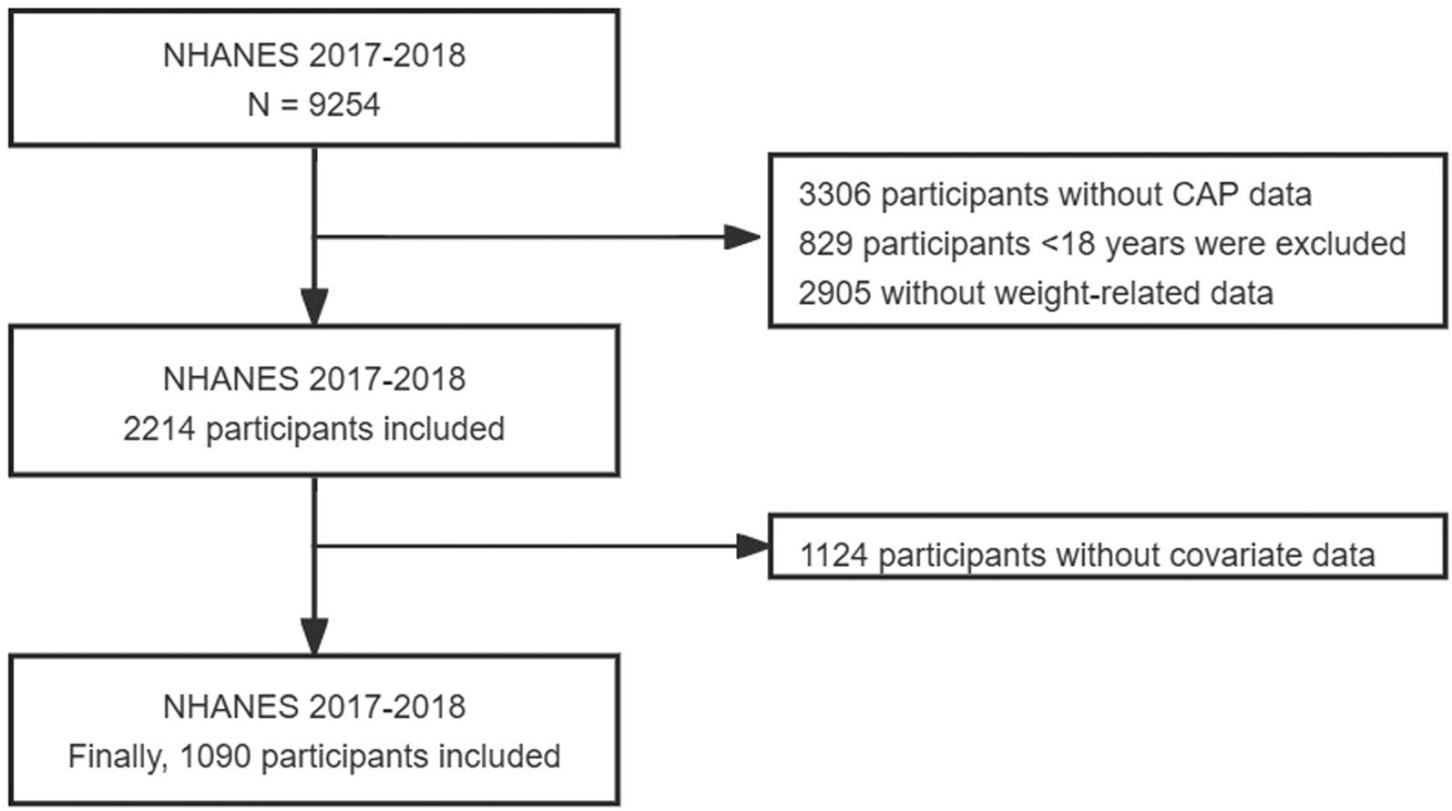
Flow chart of participants selection from the NHANES 2017–2018.

The National Health and Nutrition Examination Surveys (NHANES), which began in the early 1960s and underwent a significant transformation in 1999, is a meticulously planned study that evaluates the overall health and nutritional status of both American adults and children.^21^ In this study, we used data from NHANES 2017–2018 cycles. A total of 9254 participants were screened in this cycle according to the study objectives and methods, and 1090 participants were finally included in this study (Figure 1). The survey has received approval from the National Center for Health Statistics Research Ethics Review Committee, and informed consent has been obtained from all participants (Protocol number: 2018‐01).

### 2.2 Covariates

The variables involved in this study were all extracted from the NHANES database. These variables mainly include gender, age, race, education level, family economic status, smoking status, alcohol consumption, physical activity, past medical history, BMI, routine blood test indicators, blood lipid indicators, liver function indicators, HOMA‐IR, and so on. The diagnosis of hypertension, diabetes, and hyperlipidemia was determined based on data from questionnaires, physical examinations, and test results.^22^ Physical activity status was grouped according to PAQ questionnaire data.^23^ Smoking status and drinking consumption were determined based on survey data.^24^, ^25^ HOMA‐IR is determined by multiplying fasting insulin with fasting plasma glucose and then dividing the result by 22.5.^26^

### 2.3 Calculation of inflammatory score, other inflammatory markers, and TyG

For each participant, Z‐scores were calculated using their biomarker levels (X), the study mean (M), and the study standard deviation (SD), following the formula: z‐score = (X − M)/SD. Subsequently, the inflammatory score was determined by summing the individual Z‐scores for hs‐CRP and WBC.^9^ The following ratios were calculated: NLR as the neutrophil count divided by the lymphocyte count; MLR as the monocyte count divided by the lymphocyte count; PLR as the platelet count divided by the lymphocyte count; SII as the product of the neutrophil count and the platelet count, divided by the lymphocyte count; and SIRI as the product of the neutrophil count and the monocyte count, divided by the lymphocyte count.^27^ TyG = ln [fasting triglyceride level × fasting glucose level/2].^28^

### 2.4 Diagnosis of MAFLD

The diagnostic criteria for MAFLD align with the consensus released in 2020. When patients exhibit hepatic steatosis, a diagnosis of MAFLD will be made if they concurrently present with any one of the following conditions: overweight or obesity, type 2 diabetes, or metabolic abnormalities.^1^ Metabolic abnormalities are defined as the existence of at least two metabolic risk abnormalities in an individual. (1) Waist circumference ≥102 cm for Caucasian men and ≥88 cm for Caucasian women (or ≥90 cm for Asian men and ≥80 cm for Asian women). (2) Blood pressure ≥130/85 mmHg or specific drug treatment. (3) Plasma triglycerides ≥150 mg/dl (≥1.70 mmol/L) or specific drug treatment. (4) Plasma HDL cholesterol <40 mg/dl (<1.0 mmol/L) for men and <50 mg/dl (<1.3 mmol/L) for women, or specific drug treatment. (5) Prediabetes (i.e., fasting glucose levels of 100–125 mg/dl [5.6–6.9 mmol/L], or 2‐h postload glucose levels of 140–199 mg/dl [7.8–11.0 mmol/L], or HbA1c of 5.7%–6.4% [39–47 mmol/mol]). (6) HOMA‐IR score ≥2.5. (7) Plasma hs‐CRP level >2 mg/L. During the survey conducted from 2017 to 2018, the NHANES team used FibroScan to quantitatively assess the liver fat status of participants. The quantitative grading was determined based on the Controlled Attenuation Parameter (CAP). Based on previous research results, participants with a CAP value of ≥248 dB/m were considered to have liver steatosis.^29^

### 2.5 Statistical analysis

Given that NHANES data are constructed under a complex sampling design, the impact of weighting was considered in all relevant statistical analyses. The basic characteristics of categorical variables are presented as counts and percentages (%), while those of continuous variables are presented as medians (interquartile range). Chi‐squared tests were used to assess differences between categorical variable groups, and Mann–Whitney U tests were used for differences between continuous variable groups. Weighted logistic regression was employed to evaluate the correlation between inflammatory scores, other inflammatory markers, and MAFLD. Sensitivity analyses were conducted using propensity score matching (PSM) and unweighted multivariate logistic regression to assess the robustness of the correlation between inflammatory scores and MAFLD. We have evaluated and visualized the dose–response relationship between the inflammatory score and MAFLD using the restricted cubic splines (RCS) function from the “rms” package in R, with analysis conducted at four knots. Once the temporal relationship between inflammatory score and MAFLD was established, a mediation model was constructed to test whether the association between inflammatory score and MAFLD was mediated by TyG. In the analysis of mediation effects, the bootstrap method was employed, and the estimation and testing of mediation effects were conducted through 500 iterations of resampling.

## 3 RESULTS

### 3.1 Characteristics of participants

The final study included 1090 participants, with 563 diagnosed with MAFLD. The median age of the participants was 44 years. Participants with MAFLD exhibited significantly elevated inflammatory scores compared to those without MAFLD (0.14 [−0.63, 1.04] vs. −0.64 [−1.16, 0.08]) (Table 1). Additionally, compared to subjects without MAFLD, those with MAFLD exhibited higher levels of other inflammatory markers, including WBC, hs‐CRP, platelets, neutrophils, lymphocytes, NLR, SII, and SIRI. A *p *< .05 was considered statistically significant.

**Table 1:** Characteristic of participants.

Variables	Total (*n* = 1090)	Non‐MAFLD (*n* = 527)	MAFLD (n = 563)	*p* Value
Age (years)	44.00 (30.00, 58.00)	36.00 (25.00, 52.00)	50.00 (35.00, 62.00)	<.0001
Age group, *n*(%)				<.001
<60	809 (78.51)	427 (86.89)	382 (70.30)	
≥60	281 (21.49)	100 (13.11)	181 (29.70)	
Sex, *n*(%)				.16
Female	534 (49.06)	271 (51.51)	263 (46.65)	
Male	556 (50.94)	256 (48.49)	300 (53.35)	
BMI (kg/m²)	27.80 (23.80, 32.90)	24.40 (22.00, 27.90)	31.80 (27.80, 36.40)	<.0001
BMI category, *n*(%)				<.0001
Normal weight (<25 kg/m^2^)	329 (31.55)	279 (56.34)	50 (7.26)	
Overweight (25–30 kg/m^2^)	326 (29.17)	159 (28.08)	167 (30.25)	
Obesity (≥30 kg/m^2^)	435 (39.27)	89 (15.58)	346 (62.49)	
Waist circumference (cm)	97.40 (85.60, 110.40)	86.10 (79.80, 95.40)	107.30 (98.50, 118.40)	<.0001
Hip circumference (cm)	104.10 (97.70, 114.60)	98.90 (94.40, 106.40)	110.80 (103.50, 122.20)	<.0001
PIR, *n*(%)				.62
<1	177 (10.97)	91 (11.08)	86 (10.87)	
1–3	451 (33.54)	199 (31.46)	252 (35.57)	
>3	462 (55.49)	237 (57.46)	225 (53.56)	
Race, *n*(%)				.09
Non‐Hispanic Black	239 (10.19)	132 (11.30)	107 (9.10)	
Mexican American	154 (8.99)	53 (6.85)	101 (11.09)	
Non‐Hispanic White	390 (66.05)	179 (65.99)	211 (66.11)	
Other race	307 (14.77)	163 (15.86)	144 (13.71)	
Education levels, *n*(%)				.22
<High school	151 (7.22)	66 (6.10)	85 (8.32)	
=High school	266 (27.11)	124 (26.17)	142 (28.04)	
>High school	673 (65.66)	337 (67.72)	336 (63.64)	
Smoking status, *n*(%)				.05
Never	657 (59.76)	340 (64.20)	317 (55.42)	
Former	239 (24.29)	98 (21.24)	141 (27.27)	
Current	194 (15.95)	89 (14.56)	105 (17.31)	
Drinking status, *n*(%)				.38
Never	118 (7.16)	55 (6.48)	63 (7.82)	
Mild	483 (45.21)	235 (44.01)	248 (46.40)	
Moderate	236 (22.18)	123 (25.50)	113 (18.92)	
Heavy	253 (25.45)	114 (24.01)	139 (26.86)	
Physical activities status, *n*(%)				<.001
<150 min/week	162 (13.52)	63 (8.23)	99 (18.70)	
≥150 min/week	928 (86.48)	464 (91.77)	464 (81.30)	
DM, *n*(%)				<.0001
No	904 (87.37)	492 (96.78)	412 (78.15)	
Yes	186 (12.63)	35 (3.22)	151 (21.85)	
Hyperlipidemia, *n*(%)				<.001
No	384 (37.52)	252 (50.08)	132 (25.22)	
Yes	706 (62.48)	275 (49.92)	431 (74.78)	
Hypertension, *n*(%)				<.0001
No	677 (66.12)	400 (81.94)	277 (50.61)	
Yes	413 (33.88)	127 (18.06)	286 (49.39)	
SBP (mmHg)	119.00 (109.00, 130.00)	115.00 (105.00, 124.00)	124.00 (114.00, 134.00)	<.0001
DBP (mmHg)	72.00 (65.00, 79.00)	69.00 (63.00, 75.00)	75.00 (67.00, 81.00)	<.0001
Laboratory data				
HbA1c (%)	5.40 (5.20, 5.70)	5.30 (5.10, 5.50)	5.50 (5.30, 5.90)	<.0001
FBG (mmol/L)	5.66 (5.33, 6.11)	5.44 (5.16, 5.77)	5.88 (5.55, 6.49)	<.0001
TC (mmol/L)	4.65 (4.11, 5.40)	4.63 (4.06, 5.30)	4.76 (4.16, 5.51)	.21
HDL‐C (mmol/L)	1.37 (1.14, 1.68)	1.50 (1.24, 1.78)	1.27 (1.06, 1.53)	<.0001
TG (mmol/L)	0.95 (0.63, 1.48)	0.73 (0.54, 1.15)	1.16 (0.85, 1.72)	<.0001
LDL‐C (mmol/L)	2.77 (2.25, 3.34)	2.66 (2.20, 3.26)	2.90 (2.28, 3.47)	.14
ALT (U/L)	19.00 (14.00, 27.00)	16.00 (12.00, 23.00)	21.00 (16.00, 32.00)	<.0001
AST (U/L)	20.00 (16.00, 24.00)	20.00 (16.00, 23.00)	20.00 (16.00, 25.00)	.03
HOMA‐IR	2.21 (1.37, 4.05)	1.53 (0.96, 2.25)	3.40 (2.07, 5.67)	<.0001
Inflammatory markers				
hs‐CRP (mg/L)	1.57 (0.73, 3.72)	1.03 (0.53, 2.27)	2.30 (1.06, 4.96)	<.0001
WBC (10⁹/L)	6.40 (5.40, 7.80)	5.90 (5.00, 7.10)	7.00 (5.70, 8.40)	<.0001
Inflammatory score	−0.27 (−0.96, 0.63)	−0.64 (−1.16, 0.08)	0.14 (−0.63, 1.04)	<.0001
PLT (10⁹/L)	231.00 (199.00, 268.00)	229.00 (195.00, 261.00)	240.00 (202.00, 276.00)	.01
Neutrophils (10⁹/L)	3.60 (2.80, 4.70)	3.30 (2.50, 4.30)	4.00 (3.00, 5.00)	<.0001
Lymphocytes (10⁹/L)	2.00 (1.60, 2.40)	1.90 (1.50, 2.30)	2.10 (1.70, 2.50)	.01
Monocytes (10⁹/L)	0.50 (0.40, 0.60)	0.50 (0.40, 0.60)	0.50 (0.50, 0.60)	<.001
NLR	1.81 (1.38, 2.50)	1.74 (1.36, 2.37)	1.87 (1.41, 2.67)	.04
PLR	117.22 (94.67, 147.65)	120.91 (98.57, 152.11)	114.64 (90.42, 146.88)	.16
MLR	0.26 (0.21, 0.33)	0.27 (0.21, 0.33)	0.26 (0.21, 0.33)	.64
SII	417.52 (307.68, 606.00)	390.00 (301.28, 525.35)	448.00 (312.73, 632.67)	.01
SIRI	0.95 (0.65, 1.38)	0.83 (0.63, 1.30)	1.03 (0.71, 1.48)	<.0001

Abbreviations: ALT, alanine aminotransferase; AST, aspartate aminotransferase; BMI, body mass index; DBP, diastolic blood pressure; DM, diabetes mellitus; FBG, fasting blood glucose; HDL‐C, high‐density lipoprotein cholesterol; HOMA‐IR, homeostasis model assessment‐insulin resistance; hs‐CRP, high‐sensitivity C‐reactive protein; LDL‐C, low‐density lipoprotein cholesterol; MAFLD, metabolic associated fatty liver disease; MLR, monocyte‐to‐lymphocyte ratio; NLR, neutrophil‐to‐lymphocyte ratio; PIR, poverty income ratio; PLR, platelet‐to‐lymphocyte ratio; PLT, platelet count; SBP, systolic blood pressure; SII, systemic immune inflammation index; SIRI, system inflammation response index; TC, total cholesterol; TG, triglycerides; WBC, white blood cell.

### 3.2 The association of inflammatory score, other inflammatory markers, and the risk of MAFLD

We constructed three models for inflammatory score and other inflammatory markers respectively to assess their association with MAFLD (Table 2). Model 3 indicate that even after adjusting for potential confounding factors, the inflammatory score remains positively associated with the risk of MAFLD (odds ratio [OR] = 1.235, 95% confidence interval [CI] 1.069–1.427, *p* = .007). However, the other inflammatory markers, including NLR (OR = 1.029, 95% CI 0.849–1.248), MLR (OR = 0.303, 95% CI 0.027–3.397), PLR (OR = 0.890, 95% CI 0.237–3.342), SII (OR = 1.574, 95% CI 0.584–4.243), and SIRI (OR = 0.993, 95% CI 0.732–1.347), were no longer significantly associated with MAFLD in the Model 3 (all *p* > .05). Interestingly, in both Model 1 and Model 2, SII (Model 1, OR = 3.301, 95% CI 1.457–7.478, *p* = .007; Model 2, OR = 3.709, 95% CI 1.746‐7.877, *p* = .003) and SIRI (Model 1, OR = 1.365, 95% CI 1.094–1.701, *p* = .009; Model 2, OR = 1.311, 95% CI 1.097–1.566, *p* = .006) exhibited a positive correlation with the risk of MAFLD. However, this correlation was no longer significant in the fully adjusted model (Model 3), possibly because the true relationship between SII and SIRI with MAFLD was unveiled after adjusting for potential confounding factors.

**Table 2:** The association inflammatory score and other inflammatory markers with MAFLD.

Variables	OR	95% CI	*p* Value
Inflammatory score			
Model 1	1.571	1.333–1.851	<.0001
Model 2	1.763	1.486–2.091	<.0001
Model 3	1.235	1.069–1.427	.007
WBC			
Model 1	1.322	1.232–1.419	<.0001
Model 2	1.420	1.299–1.551	<.0001
Model 3	1.128	1.006–1.266	.041
hs‐CRP			
Model 1	1.124	1.027–1.231	.015
Model 2	1.162	1.048–1.288	.008
Model 3	1.042	1.002–1.083	.039
NLR			
Model 1	1.182	0.974–1.434	.085
Model 2	1.114	0.952–1.304	.161
Model 3	1.029	0.849–1.248	.753
MLR			
Model 1	0.706	0.145–3.427	.644
Model 2	0.199	0.052–0.758	.022
Model 3	0.303	0.027–3.397	.309
PLR			
Model 1	0.325	0.072–1.466	.132
Model 2	0.325	0.053–0.722	.018
Model 3	0.890	0.237–3.342	.854
SII			
Model 1	3.301	1.457–7.478	.007
Model 2	3.709	1.746–7.877	.003
Model 3	1.574	0.584–4.243	.345
SIRI			
Model 1	1.365	1.094–1.701	.009
Model 2	1.311	1.097–1.566	.006
Model 3	0.993	0.732–1.347	.961

*Note*: Model 1 was the crude model; Model 2 was adjusted for sex and age; Model 3 was adjusted for sex, age, race, education level, PIR, BMI, smoking status, drinking status, hyperlipidemia, hypertension, DM, physical activities status, ALT, and AST.

Abbreviations: ALT, alanine aminotransferase; AST, aspartate aminotransferase; BMI, body mass index; CI, confidence interval; DM, diabetes mellitus; hs‐CRP, high‐sensitivity C‐reactive protein; MAFLD, metabolic associated fatty liver disease; MLR, monocyte‐to‐lymphocyte ratio; NLR, neutrophil‐to‐lymphocyte ratio; OR, odds ratio; PIR, poverty income ratio; PLR, platelet‐to‐lymphocyte ratio; SII, systemic immune inflammation index; SIRI, system inflammation response index; WBC, white blood cell.

As mentioned earlier, the sensitivity analysis was conducted based on PSM and unweighted logistic regression. The results showed that in the Model 3, the inflammatory score remains positively associated with MAFLD (unweighted logistic regression OR = 1.168, 95% CI 1.047–1.302, *p* = .005; and weighted logistic regression after PSM OR = 1.240, 95% CI 1.031–1.492, *p* = .025, respectively). These findings align with the initial results, indicating a reliable positive association between inflammatory score and MAFLD, as detailed in Table 3. After PSM, the comparison of demographic data between the non‐MAFLD and MAFLD groups can be found in Supporting Information: Table S1.

**Table 3:** Sensitivity analysis of the association between inflammatory score and MAFLD based on unweighted logistic regression and PSM.

Inflammatory score	Unweighted logistic regression		PSM	
	OR (95% CI)	*p* Value	OR (95% CI)	*p* Value
Model 1	1.473 (1.331–1.630)	<.0001	1.788 (1.518–2.107)	<.0001
Model 2	1.587 (1.426–1.768)	<.0001	1.901 (1.622–2.227)	<.0001
Model 3	1.168 (1.047–1.302)	.005	1.240 (1.031–1.492)	.025

*Note*: Model 1 was the crude model; Model 2 was adjusted for sex and age; Model 3 was adjusted for sex, age, race, education level, PIR, BMI, smoking status, drinking status, hyperlipidemia, hypertension, DM, physical activities status, ALT, and AST.

Abbreviations: ALT, alanine aminotransferase; AST, aspartate aminotransferase; BMI, body mass index; CI, confidence interval; DM, diabetes mellitus; MAFLD, metabolic associated fatty liver disease; OR, odds ratio; PIR, poverty income ratio; PSM, propensity score matching.

### 3.3 Investigating the dose–response relationship between inflammatory score and MAFLD

**Figure 2:**
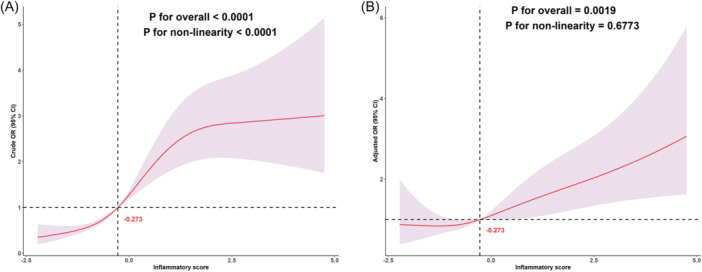
Dose–response relationship between inflammatory score and MAFLD. (A) Unadjusted dose–response relationship between inflammatory score and MAFLD; (B) Adjusted dose–response relationship between inflammatory score and MAFLD. Adjusted factors include sex, age, race, education level, PIR, BMI, smoking status, drinking status, hyperlipidemia, hypertension, DM, physical activities status, ALT, and AST. *p* for over‐all <.05 indicates that the association between the inflammatory score and MAFLD is significant, while *p* for nonlinearity <.05 suggests that the dose–response relationship between the inflammatory score and MAFLD is nonlinear; conversely, it is linear. ALT, alanine aminotransferase; AST, aspartate aminotransferase; BMI, body mass index; DM, diabetes mellitus; MAFLD, metabolic associated fatty liver disease; PIR, poverty income ratio.

Figure 2 presents the results of the dose–response relationship between inflammatory score and MAFLD based on RCS. The results indicate that before adjusting for confounding factors, there is a nonlinear dose–response relationship between inflammatory score and MAFLD (*p* for nonlinearity < .0001). However, after adjusting for potential confounding factors(same as Model 3), the true dose–response relationship pattern between inflammatory score and MAFLD emerges, showing a linear dose–response relationship (*p* for nonlinearity = .6773), indicating that the risk of MAFLD increases with a higher inflammatory score.

### 3.4 The mediating effect of IR in the association between inflammatory score and MAFLD

**Figure 3:**
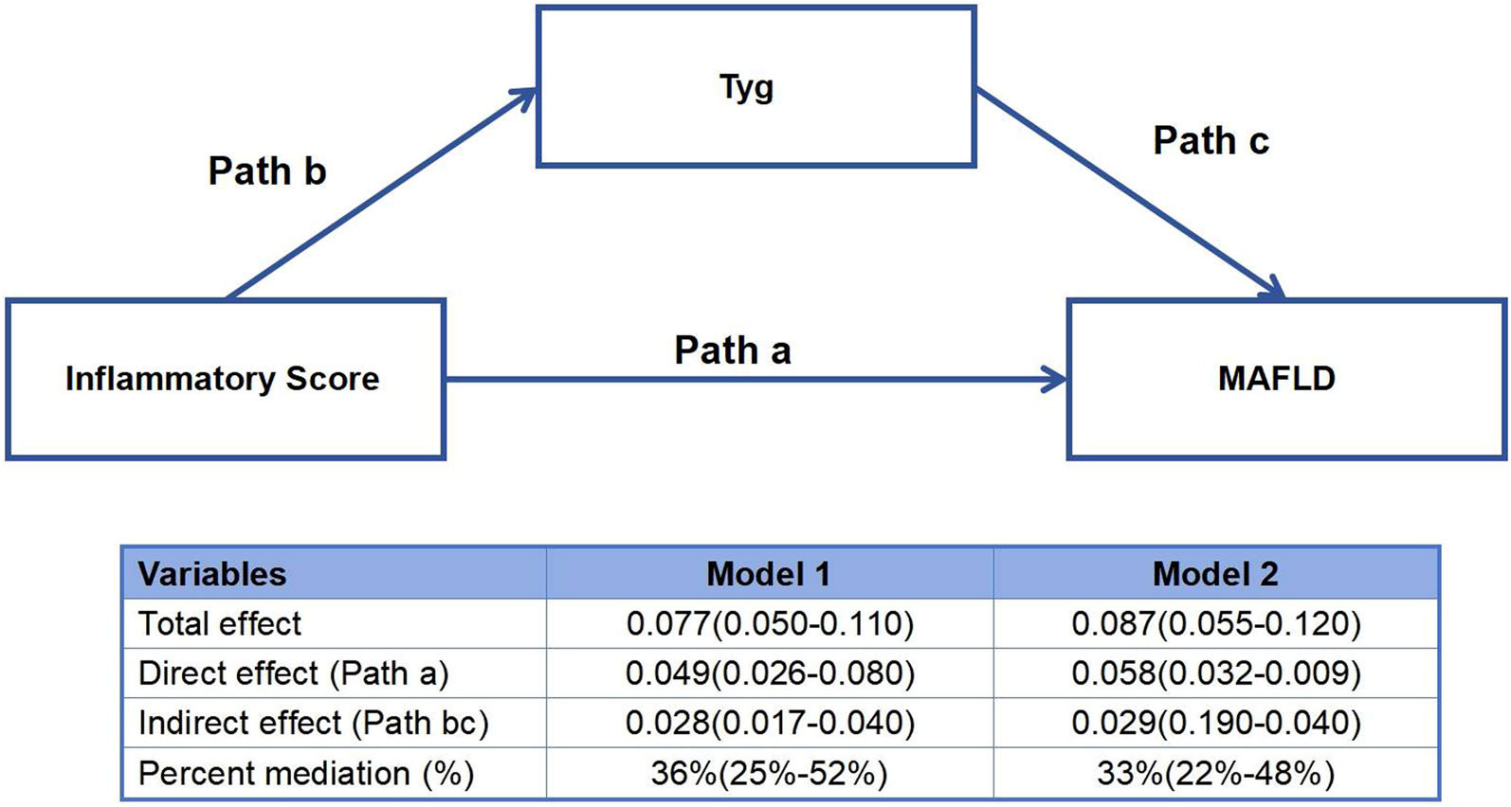
Mediation analysis for Tyg in the association between Inflammatory score and MAFLD. Total effect: the overall impact of inflammatory score (X) on MAFLD (Y), without considering the mediating effect of insulin resistance (M, evaluated by TyG); Direct effect: the direct impact of inflammatory score (X) on MAFLD (Y) after controlling for the effect of insulin resistance (M, evaluated by TyG); Indirect effect: the indirect impact of inflammatory score (X) on MAFLD (Y) through insulin resistance (M, evaluated by TyG); Percent mediation: the proportion of the indirect effect in the total effect, reflecting the importance of insulin resistance (M, evaluated by TyG) in the relationship between inflammatory score (X) and MAFLD (Y). Model 1 was the crude model; Model 2 was adjusted for sex and age. MAFLD, metabolic associated fatty liver disease; TyG, triglyceride‐glucose.

In this mediation analysis, we considered TyG as a mediator variable to investigate whether and to what extent it mediated the association between inflammatory score and MAFLD. Mediation analysis showed that 36% of the association between inflammatory score and the risk of MAFLD may be mediated by TyG, and this mediation effect persisted even after adjusting for sex and age factors, with a percent mediation of 33% (Figure 3).

## 4 DISCUSSION

We derived an inflammatory score using hs‐CRP and WBC to represent the systemic inflammatory level in this cross‐sectional study. Both multivariable logistic regression and RCS analyses demonstrated a strong positive association between the inflammatory level and the presence of MAFLD. Additionally, our study found that TyG, a reliable surrogate marker of IR, mediates the association between inflammation and the prevalence of MAFLD, providing a new insight for understanding the potential association between inflammation, IR, and MAFLD.

Unlike NAFLD, MAFLD emphasizes the role of metabolic abnormalities in the occurrence and development of the disease. Multiple studies have confirmed the association between WBC, hs‐CRP, and metabolic abnormalities,^30^, ^31^ while research by Jung et al.^32^ further suggests a causal relationship between WBC and the future development of metabolic syndrome in healthy adults at baseline. Similarly, hs‐CRP is considered a marker of chronic low‐grade inflammation, and patients with metabolic syndrome often exhibit a chronic low‐grade inflammatory state, which increases their risk of developing diabetes and cardiovascular events in the future.^32^ Additionally, Ford et al.^33^ found a positive association between hs‐CRP levels and the number of components of metabolic syndrome, indicating that as hs‐CRP levels increase, individuals are more likely to have multiple metabolic abnormalities, and other studies have reached similar conclusions.^34^, ^35^, ^36^, ^37^ Some studies also suggest a strong association between WBC, hs‐CRP, and metabolic syndrome, diabetes, and cardiovascular health.^9^, ^10^ It's worth noting that although our study also found the association between WBC, hs‐CRP, and MAFLD, similar to previous research,^27^, ^38^ our results indicate that the association between WBC, hs‐CRP, and MAFLD is weaker than that between the inflammatory score and MAFLD. This may be because the inflammatory score derived from WBC to hs‐CRP can better reflect the systemic inflammatory level of the body compared to individual inflammatory markers and can also reflect the state of metabolic abnormalities to some extent. As mentioned earlier, inflammation and metabolic abnormalities play crucial roles in the pathogenesis of MAFLD, which may be one of the reasons for the strong association between the inflammatory score and MAFLD.

However, other composite inflammatory markers were not found to be associated with MAFLD in our study, consistent with some previous research.^39^, ^40^, ^41^ We hypothesize that this could be explained by genetic studies that have confirmed no causal association between neutrophils, lymphocytes, monocytes, and type 2 diabetes,^42^ which may partially explain the insignificant correlation between composite inflammatory markers and MAFLD in our study. Additionally, these composite inflammatory markers represent different inflammatory pathways. Our previous research has shown that NLR, PLR, SII, and SIRI indicate the inflammatory level under stress and are associated with adverse outcomes.^43^, ^44^ The pathogenesis of MAFLD may be more inclined toward the continuous effect of chronic inflammation.^15^, ^45^, ^46^ Although no association between these composite inflammatory markers and MAFLD was found in our study, some studies came to the opposite conclusion.^27^, ^40^, ^47^ For instance, while Liu et al.'s study found a positive correlation between PLR and SIRI with MAFLD, our research found no significant correlation between PLR and SIRI with MAFLD. This disparity may stem from the distinct populations included in the two studies. Specifically, Liu et al.'s study primarily enrolled a general Chinese population aged 35–75 years. Additionally, there were differences in the confounding factors adjusted between the two studies. These factors could all contribute to the variation in results. From our perspective, discrepancies in research design, sample specificity, sample size, statistical methods, and adjusted confounding factors among different studies can all lead to differences in outcomes. In conclusion, the potential association between composite inflammatory markers and MAFLD requires further investigation.

As previously mentioned, the inflammatory response is intertwined with the entire process of the occurrence and development of MAFLD. The accumulation of liver fat and excess free fatty acids can lead to damage to hepatocytes, accompanied by oxidative stress, endothelial dysfunction, and increased secretion of inflammatory factors.^46^ Certain inflammatory factors can directly interfere with the signaling pathway through which insulin exerts its biological effects, exacerbating IR,^48^, ^49^ and IR is typically associated with a systemic low‐grade inflammatory state.^50^, ^51^ Although the relationship between inflammation, IR, and MAFLD has long been established, few studies have quantified the association among these three factors. In our study, we evaluated patients' systemic inflammatory burden based on the inflammatory score, used TyG to assess the body's IR status, and quantified the relationship among the three based on mediation analysis. The final results indicated that approximately 33% of the effect of inflammation on MAFLD is mediated by TyG. This finding further clarifies the intrinsic relationship among inflammation, IR, and MAFLD, emphasizing the importance of improving IR and reducing the body's inflammatory level to reduce the risk of MAFLD. It is worth mentioning that this study only explored the association between inflammatory score and MAFLD. Future research could further investigate whether there is a similar positive correlation between inflammatory score and metabolic dysfunction‐associated steatotic liver disease (MASLD). This may provide additional evidence to confirm the potential association between inflammatory score and this category of diseases.

## 5 STRENGTHS AND LIMITATIONS

Our study has several advantages: First, the comprehensive data collection in NHANES allowed us to thoroughly adjust for potential confounders when assessing the correlation between the inflammatory score and MAFLD. Additionally, we employed the CAP to evaluate hepatic steatosis, which, although not as precise as liver biopsy, has gained widespread recognition for its accuracy. Most importantly, our study is the first to uncover a dose–response relationship between the inflammatory score and MAFLD, as well as to elucidate the intrinsic links among inflammation, IR, and MAFLD. However, this study also has the following limitations: First, the inflammatory score possesses sample specificity, which means the inflammation score calculated in this study may only be applicable to the American population. Second, as a cross‐sectional study, it is inherently limited and unable to establish a causal relationship between the inflammatory score and MAFLD. Additionally, it is necessary to explore the association between the inflammatory score and MASLD in future studies. Finally, the diagnosis of hepatic steatosis did not utilize biopsy, which is considered the gold standard.

## 6 CONCLUSION

Our research findings indicate a strong association between inflammatory score and the risk of MAFLD. Mediation analysis revealed that 33% of the association was mediated by TyG. This provides a new insight for understanding the potential relationship between inflammation, IR, and MAFLD. This discovery underscores the importance of improving inflammatory levels and IR in reducing the risk of MAFLD.

## AUTHOR CONTRIBUTIONS

**Yan Chen**: Conceptualization; methodology; data curation; visualization; writing—original draft; writing—review and editing. **Xin Zhao**: Conceptualization; funding acquisition; supervision.

## CONFLICT OF INTEREST STATEMENT

The authors declare no conflict of interest.

## ETHICS STATEMENT

The survey were approved by the Ethics Review Board of the National Center for Health Statistics (Protocol number: 2018‐01) and were in accordance with the principles of the Declaration of Helsinki. Informed consent was obtained from all participants.

## Supporting information

Supporting information.

## Data Availability

Data associated with this study are freely available from the NHANES website (www.cdc.gov/nchs/nhanes/).
