# Supplementary material for: The mediating role of insulin resistance in the association between inflammatory score and MAFLD: NHANES 2017–2018
Source: Immun Inflamm Dis. 2024 Oct 4;12(10):e70035. doi: 10.1002/iid3.70035 (PMC11450453; doi:10.1002/iid3.70035)
Supplement: Supplementary file 1 — Supporting information. [file IID3-12-e70035-s001.docx]

**Supplementary Table 1 Comparison of demographic characteristics between the two groups after PSM**

| **Variable** | **Without MAFLD(N=427)** | **MAFLD(N=427)** | ***P*** |
| --- | --- | --- | --- |
| Age (years) | 43.00(29.00,56.00) | 46.00(33.00,60.00) | 0.19 |
| Sex, n (%) |  |  | 0.99 |
| Female | 194(49.30) | 198(49.36) |  |
| Male | 209(50.70) | 205(50.64) |  |
| RACE,n (%) |  |  | 0.09 |
| Non-Hispanic Black | 109(12.93) | 78( 9.57) |  |
| Mexican American | 41(6.83) | 73(11.69) |  |
| Non-Hispanic White | 131(65.99) | 147(65.41) |  |
| Other Race | 122(14.25) | 105(13.33) |  |
| Education levels,n (%) |  |  | 0.77 |
| <high school | 59(7.84) | 48(6.56) |  |
| =high school | 94(26.57) | 103(26.33) |  |
| >high school | 250(65.59) | 252(67.11) |  |
| PIR, n (%) |  |  | 0.84 |
| ≤1 | 74(11.75) | 59(10.76) |  |
| 1-3 | 157(34.05) | 190(36.39) |  |
| >3 | 172(54.20) | 154(52.85) |  |

Abbreviations: PSM, propensity score matching; MAFLD, metabolic associated fatty liver disease; PIR, poverty income ratio.
